# Reliable Acoustic Path and Direct-Arrival Zone Spatial Gain Analysis for a Vertical Line Array

**DOI:** 10.3390/s18103462

**Published:** 2018-10-15

**Authors:** Chunyu Qiu, Shuqing Ma, Yu Chen, Zhou Meng, Jianfei Wang

**Affiliations:** College of Meteorology and Oceanology, National University of Defense Technology, Changsha 410073; China; qiuchunyu12@nudt.edu.cn (C.Q.); chenyulm@163.com (Y.C.); mengzhou@nudt.edu.cn (Z.M.); wjfjoy@126.com (J.W.)

**Keywords:** vertical line array, spatial gain, correlation, vertical directionality, direct-arrival zone, reliable acoustic path, deep ocean, ray theory, multiple effect

## Abstract

A method is developed in this paper to calculate the spatial gain of a vertical line array when the plane-wave assumption is not applicable and when the oceanic ambient noise is correlated. The proposed optimal array gain (OAG), which can evaluate the array’s performance and effectively guide its deployment, can be given by an equation in which the noise gain (NG) is subtracted from the signal gain (SG); hence, a high SG and a negative NG can enhance the performance of the array. OAGs and SGs with different array locations are simulated and analyzed based on the sound propagation properties of the direct-arrival zone (DAZ) and the reliable acoustic path (RAP) using ray theory. SG and NG are related to the correlation coefficients of the signals and noise, respectively, and the vertical correlation is determined by the structures of the multipath arrivals. The SG in the DAZ is always high because there is little difference between the multipath waves, while the SG in the RAP changes with the source-receiver range because of the variety of structure in the multiple arrivals. The SG under different conditions is simulated in this work. The “dual peak” structure can often be observed in the vertical directionality pattern of the noise because of the presence of bottom reflection and deep sound channel. When the directions of the signal and noise are close, the conventional beamformer will enhance the correlation of not only the signals but also the noise; thus, the directivity of the signals and noise are analyzed. Under the condition of having a typical sound speed profile, the OAG in some areas of the DAZ and RAP can achieve high values and even exceed the ideal gain of horizontal line array 10 *l*og*N* dB, while, in some other areas, it will be lowered because of the influence of the NG. The proposed method of gain analysis can provide analysis methods for vertical arrays in the deep ocean under many conditions with references. The theory and simulation are tested by experimental data.

## 1. Introduction

The direct-arrival zone (DAZ) and reliable acoustic path (RAP) are important acoustic ducts in deep water. Gain analysis based on the DAZ and RAP is essential for evaluating the detection performance of the vertical array and for guiding its deployment.

In recent years, applications of the DAZ and RAP have received increasing attention because of their unique acoustic properties. In the DAZ, sound signals arrive at the hydrophone mainly through direct and surface-reflected ray paths [1]. The propagation properties of the DAZ were presented in [1,2] but have not been applied to correlation or gain analysis. The acoustic properties of the RAP were researched and applied for source localization [3]. Reference [4] reported measurements and analysis on vertical correlations of signals received by an array deployed in the RAP under a fixed source-receiver range.

In comparison with a horizontal line array, the deployment of a vertical array is an easier way to detect underwater targets in the deep ocean. The array gain is a key factor for a sonar system. A concern that arises when considering a deep ocean, which is range independent, is that the spatial gain of the vertical array is influenced by the signal distortion that results from the multipath effect and the vertical correlation of the noise [5,6]. If it is assumed that the incident signal is a plane wave and the noise from the array elements are uncorrelated, the gain of the vertical array can be calculated with a simple equation. However, because the above two assumptions are difficult to achieve, the spatial gain and its variation must be estimated properly. In [5,7], the approach to calculating the spatial gain is proposed when the arrival signals at the vertical array are not plane waves but the ambient noise is assumed to be Gaussian white. In fact, the noise constituents of different elements are not independent in the real ocean. The correlation and directivity of the noise are also important factors to consider in gain analysis. The most famous theoretical model for ambient noise was developed by Cron and Sherman in a semi-infinite homogeneous ocean [8]. Cox gave a further result that applied to this type of noise field [9]. Various approximations commonly made in noise and noise coherence models were investigated in [10] and it shown that in many cases a very simple ray approach can produce the same answers as full wave treatments. Buckingham developed theoretical models for vertical directionality and spatial coherence based on a uniform distribution of wind-generated ambient noise surface sources in a semifinite homogenous ocean [11,12]. A ray approach and parabolic equation solution were utilized to model the ambient noise field in the deep ocean by considering their calculation accuracy and efficiency in near-field wind-driven and far-field distant shipping noise fields [13]. The spatial coherence functions and directivity of shipping noise are derived in [14]. A ray-based noise model is developed to calculate the two-point spatial coherence function for ocean-surface-generated noise using a ray-tracing model in [15], it derived a Maclaurin series of the formulated spatial coherence function which explicitly revealed the feature of spatial coherence for the noise field as a function of the orientation of the hydrophone pair and the ocean environmental coefficients. The correlation and directivity of noise have been researched a large amount but are seldom applied for gain analysis.

In this paper, the spatial array gain of a vertical line array in the DAZ and RAP are analyzed based on conventional beamformer and ray theory. The different noise contributions in each of the array elements are correlated and the received signals are affected by multipath effect. The proposed optimal array gain (OAG), which can evaluate the array’s performance and guide the deployment of it effectively, can be given by an equation in which the noise gain (NG) is subtracted from the signal gain (SG). SG and NG are related to the vertical correlation coefficients of the signals and noise, respectively. The SG under various circumstances and the OAG with different array locations are simulated and analyzed. The simulation results were consistent with the experimental results measured in the South China Sea.

This paper is organized as follows: in Section 2, the propagation properties of the acoustic field in the DAZ and RAP are studied based on ray theory. The definitions and calculation methods of SG, NG and OAG are given in Section 3. Section 4 presents the results on the SG by analyzing the correlations of the signals, and it introduces the correlation and directivity of the noise. In Section 5, the OAGs for different array locations are researched by combining the properties of the signals and noise associated with each signal. The analysis is partly validated with data from a deep ocean experiment in Section 6. Section 7 summarizes the discussion and conclusions.

## 2. The Direct-Arrival Zone and the Reliable Acoustic Path

**Figure 1:**
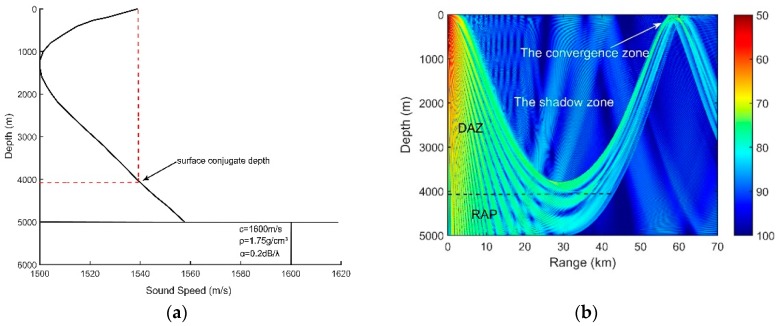
(**a**) Munk sound speed profile; (**b**) Transmission loss for a source at 100 m depth at the frequency of 150 Hz.

The sound speed profile (SSP) presented in Figure 1a is a typical deep ocean profile. The ocean depth is 5000 m. The sound speed, density and attenuation of the basement are 1600 m/s, 1.75 g/${\mathrm{cm}}^{3}$ and 0.2 dB/λ, respectively. The deep sound channel (DSC) depth and surface conjugate depth were approximately 1200 m and 4050 m, respectively. Our simulation results of the transmission loss and ray structures are implemented using the BELLHOP ray tracing model. In the deep ocean, most of the targets are shallow sources. Corresponding to the parameters above, therefore, the transmission loss of the sound pressure at 150 Hz for a source at a 100-m depth is calculated by the ray model and is shown in Figure 1b. The direct-arrival zone (DAZ), the reliable acoustic path (RAP), the shadow zone and the convergence zone can be seen in Figure 1b.

### 2.1. The Direct-Arrival Zone

**Figure 2:**
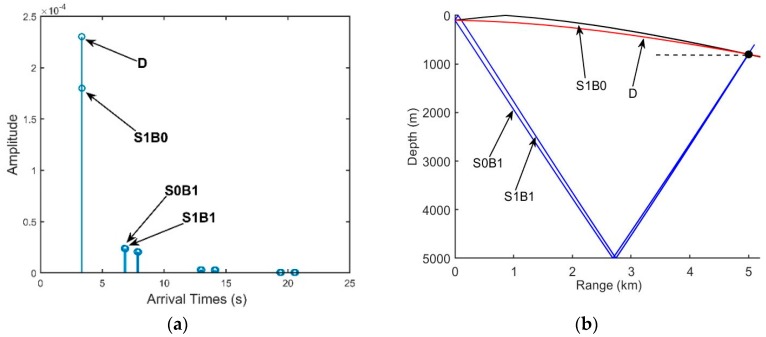
(**a**) Arrival times and amplitudes of the eigenrays and (**b**) Propagation paths of the eigenrays.

The DAZ is an important acoustic duct in the deep ocean. As shown in Figure 1b, the DAZ is always close, covering several kilometers due to the water refraction depending on the gradient of the near-surface SSP and the source depth, and in addition, its range becomes wider with the increase in the depth [1]. We consider a 16-element vertical line array with an equal spacing of 5 m. It is at a depth of 800 m. The signal source is located at a 3.1-km range and is at a depth of 100 m. The eigenray arrival structures of the first hydrophone are presented in Figure 2. Figure 2a shows clearly that the powers of the direct ray (D) and the once-surface-reflected ray (S1B0) are much larger than those of others such as the once-bottom-bounce ray (S0B1). Therefore, the multipath arrivals of the received signals in the DAZ are dominated by D and S1B0, which in fact determine the interference characteristics of the acoustic field. Other rays that undergo bottom bounces with large grazing angles can be neglected due to serious sound attenuation. As shown intuitively in Figure 1b, steady interference stripes that stem from the Lloyd-mirror effect [16] can be seen in the DAZ.

**Figure 3:**
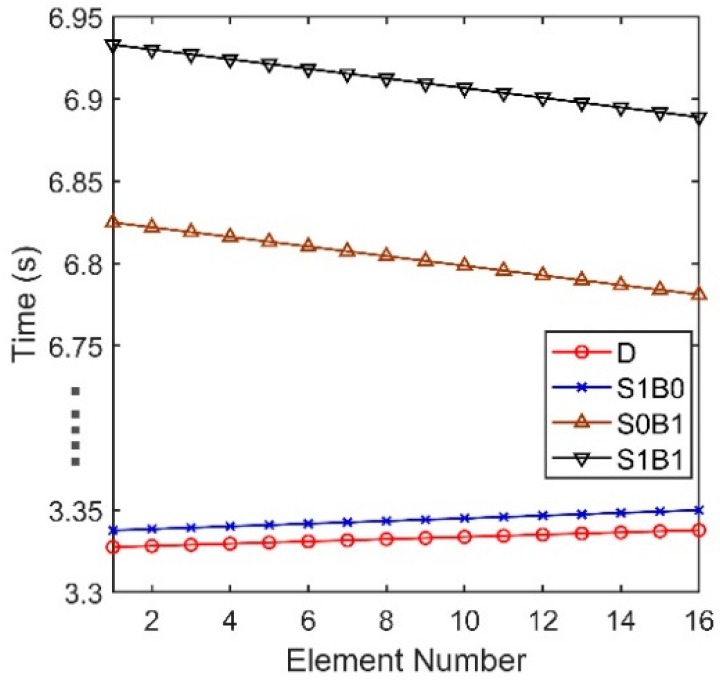
Arrival times of D, S1B0, S0B1 and S1B1.

We define that the positive and negative angles represent the downward and upward acoustic rays arriving at the hydrophone, respectively. From Figure 2b, the arrival angles of D and S1B0 are approximately 12°, while the bottom reflected rays are less than −50°. Figure 3 shows the arrival times of D, S1B0, S0B1 and S1B1 in all elements. For a long range, the spherical wave from the point source moves like a plane wave, hence the four multiple waves can be viewed as plane waves respectively. The arrival times of D and S1B0 are both almost proportional to the element number, and they have almost the same deviation between two neighboring elements. The results in Figure 2b and Figure 3 indicate that the arrival angles of D and S1B0 are approximately equal and there is little difference between these two multipath waves. Therefore, D and S1B0 can be viewed approximately as two parallel plane waves if the array aperture is not large, hence their phase difference between two hydrophones will be almost same. In contrast, the signals of S0B1 and S1B1 arrive at the last element first. The cause is that their eigenrays are refracted and reflected. The equiphase surfaces of D and S0B1 are not parallel, and hence it results in signal’s phase distortion. If S0B1 and S1B1 are also significant in an acoustic field, a conventional beamformer (CBF) will aggravate the wave distortion derived from S0B1 and S1B1. While in DAZ, the acoustic signal is almost dominated by D and S1B0, and thus, the CBF that applies the proper phase shift can almost eliminate the distortion and provide a relatively high correlation between the signals of any two hydrophones.

**Figure 4:**
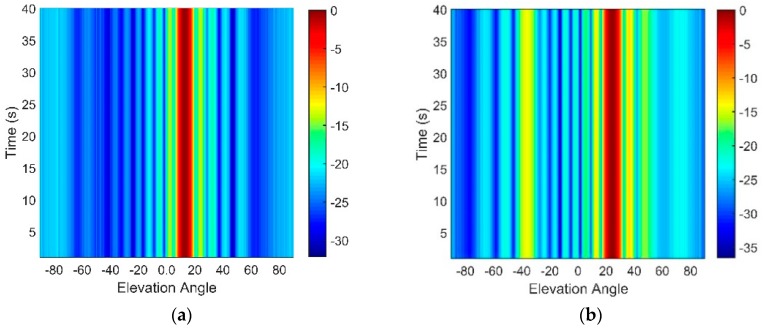
Beam output of a vertical line array at a depth of (**a**) 800 m and (**b**) 3800 m.

By beamforming, the beam intensity output with a 0.2° sampling interval is shown in Figure 4. In Figure 4a, the depth of the array is 800 m, and it is relatively small. There is almost no beam-splitting due to having only a small difference in the multipath arrival angles. While in Figure 3b, the array is at the depth of 3800 m, where the destructive interference between D and S1B0 appear. Other than D and S1B0, S0B1 also contributes to the sound field here. A splitting with two elevation angles can be observed at this depth due to multipath propagation, and it causes a lower beamforming rate.

Two big advantages of the sound transmission under DAZ environmental conditions are the simple multipath arrival structure and the low transmission loss.

### 2.2. The Reliable Acoustic Path

When the hydrophones are placed below the surface conjugate depth (the critical depth), the propagation path of D between the source and the received hydrophones is called the RAP. As shown in Figure 1b, for a source within the middle range (approximately 30 km here), the region below the conjugate depth is zoned as RAP. It is a special acoustic duct in deep water and is known to have three advantages [17]. First, the RAP is the direct path between the source and the receiver, and thus, it is insensitive to the surface scattering and the bottom reflection loss. The transmission loss that corresponds to the RAP is much lower than the loss that corresponds to the other ducts, such as the surface duct and DSC. The ambient noise that accompanies the RAP is much lower than the average ambient noise in the deep ocean. The main reason is that the distant shipping noise cannot penetrate to a depth below the critical depth according to Snell’s law. Hence, the *SNR*s of the received sound pressure in the RAP can be very high. Second, the RAP has good stability in the time/space/frequency domain [4], and the SSP fluctuations and surface scattering have little effect on its acoustic propagation. The major reason is that the D received in RAP has a relatively large grazing angle. According to Snell’s law, the fluctuation of the SSP has little impact on the refraction angle of a ray with a large grazing angle. Third, compared to the surface duct and the DSC where the sound propagation always suffers from the shadow zone, the sensors located in the RAP can detect the surface target and the submerged target in the medium range without having any blind zone.

## 3. Calculation of the Array Spatial Gain

### 3.1. Definition of the Vertical Correlation Coefficient

The normalized cross-spectrum density is an effective method for analyzing the correlation of sound fields in the frequency domain. In real underwater acoustic signal processing and detection, it is impossible to preset the frequencies of the signals and the noise associated with each signal. Usually, a relatively wide working bandwidth will be selected during the preliminary processing. The spatial correlation coefficients and the method of spatial gain analysis based on time-domain signals will be introduced in this section.

For a range-independent acoustic field, its space-time correlation function is defined as:

(1) $$\varphi_{12}\left(\tau ,r\right)=\underset{T\to \infty }{\lim}\frac{1}{T}\int_{-\frac{T}{2}}^{\frac{T}{2}}p_{1}\left(t,0\right)p_{2}^{*}\left(t+\tau ,r\right)dt$$

where $p_{1}$ and $p_{2}$ are the complex pressures on two hydrophones at different locations. Here, ∗ stands for complex conjugation, r denotes the position vector, and τ is the time delay.

For two hydrophones that are distributed vertically at a given time, Equation (1) can be simplified as:

(2) $$\varphi_{12}\left(0,\Delta z\right)=\underset{T\to \infty }{\lim}\frac{1}{T}\int_{-\frac{T}{2}}^{\frac{T}{2}}p_{1}\left(t,z\right)p_{2}^{*}\left(t,z+\Delta z\right)dt=\bar{p_{1}\left(t,z\right)p_{2}^{*}\left(t,z+\Delta z\right)}$$

where the bar over the numerator denotes the time average. Hence, the normalized vertical correlation function is given by:

(3) $$\rho_{12}\left(z,z+\Delta z\right)=\frac{Re\left[\varphi_{12}\left(\Delta z\right)\right]}{\sqrt{\varphi_{11}\left(z\right)\varphi_{22}\left(z+\Delta z\right)}}$$

### 3.2. Calculation of the Array Gain Based on the Correlations of the Acoustic Field

The array gain is defined as the improvement in the output *SNR* over the input *SNR*:

(4) $$AG=10\;lg\left(\frac{SNr_{out}}{SNr_{in}}\right)$$

When the plane-wave model assumption and isotropic Gaussian noise assumption (the noise contributions of the array elements are independent, and the signal is unrelated to the noise) are made, a conventional beamformer that applies the “delay-sum-square” model can be used to calculate the optimal array gain. The gain of the system after beamforming can achieve 10 *logN* dB, where *N* denotes the element number. However, the two assumptions above are never set up in a real ocean.

Considering a vertical line array of *M* elements with the signal $s_{u}\left(t\right)$ and noise $n_{u}\left(t\right)$ at the *u*th element, the average *SNR* at the output of the array will be:

(5) $$\frac{\bar{S^{2}}}{\bar{N^{2}}}=\frac{\bar{{\left[\sum_{u=1}^{M}s_{u}\left(t\right)\right]}^{2}}}{\bar{{\left[\sum_{u=1}^{M}n_{u}\left(t\right)\right]}^{2}}}$$

Equation (5) can be written as:

(6) $$\frac{\bar{S^{2}}}{\bar{N^{2}}}=\frac{\sum_{u}^{M}\sum_{v}^{M}\bar{s_{u}s_{v}}}{\sum_{u}^{M}\sum_{v}^{M}\bar{n_{u}n_{v}}}$$

The oceanic noise can be viewed as a stationary random process within a certain amount of time. In common cases, both the signal and the noise have approximately the same amplitude at each hydrophone, *s* and *n*, respectively. We define:

(7) $$\bar{s_{1}{}^{2}}=\bar{s_{2}{}^{2}}=\cdots =\bar{s_{M}{}^{2}}=\bar{s^{2}}$$

(8) $$\bar{n_{1}{}^{2}}=\bar{n_{2}{}^{2}}=\cdots =\bar{n_{M}{}^{2}}=\bar{n^{2}}$$

Equation (4) can be simplified to:

(9) $$\frac{\bar{S^{2}}}{\bar{N^{2}}}=\frac{\bar{s^{2}}}{\bar{n^{2}}}\frac{\sum_{u}^{M}\sum_{v}^{M}{\left(\rho_{s}\right)}_{uv}}{\sum_{u}^{M}\sum_{v}^{M}{\left(\rho_{n}\right)}_{uv}}$$

$\sum_{u}^{M}\sum_{v}^{M}{\left(c_{s}\right)}_{uv}$ denotes the sum of the correlation coefficients between all pairs of hydrophones in the signal field, and $\sum_{u}^{M}\sum_{v}^{M}{\left(c_{n}\right)}_{uv}$ is the same measure in the noise field. We have chosen to take the $\bar{s^{2}}$-to-$\bar{n^{2}}$ ratio as the “input *SNR*” reference required in Equation (4).

Accordingly, AG is defined as:

(10) $$AG=10\;lg\frac{\bar{S^{2}}/\bar{N^{2}}}{\bar{s^{2}}/\bar{n^{2}}}=10\;lg\frac{\sum_{u}^{M}\sum_{v}^{M}{\left(\rho_{s}\right)}_{uv}}{\sum_{u}^{M}\sum_{v}^{M}{\left(\rho_{n}\right)}_{uv}}$$

Simple addition of hydrophone voltages forms a beam perpendicular to the array, while in general circumstances the directionality of signal will not be perpendicular to the array. Hence, the AG derived from Equation (10) usually does not make sense. In this paper, to achieve the optimal array gain (OAG) that can evaluate the performance of vertical line array and guide the deployment of it effectively, CBF is applied as a preprocessing tool for the received signals.

**Figure 5:**
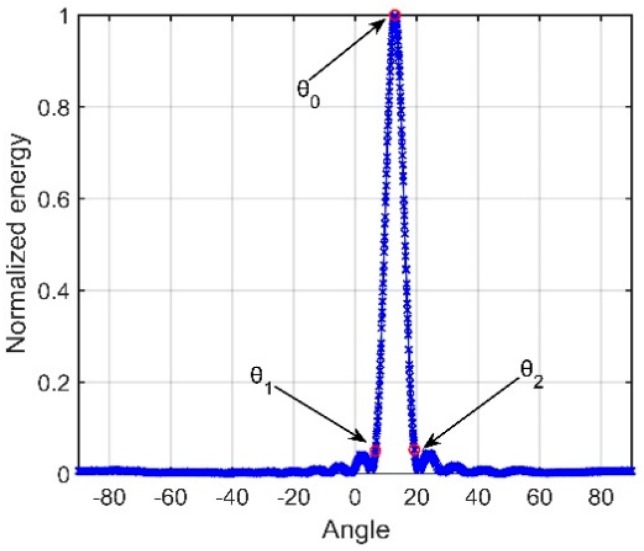
Beam output of the vertical line array.

We define the steering angle that corresponds to the maximum angular response of the array gain (AG) as $\theta_{0}$. Overall, $\theta_{0}$ would be within the range of the beam width ($\theta_{1}$~$\theta_{2}$) shown in Figure 5.

The received signals can be viewed approximately as two parallel plane waves. We define the arrival angles of D and S1B0 as $\theta_{d}$ and $\theta_{s}$, respectively (the angle that corresponds to the maximum output power of CBF can, in general, be considered $\theta_{d}$). In most of the space of the DAZ, the following will be true:

(11) $$\theta_{d}\approx \theta_{s}\approx \theta_{0}$$

The received signal and noise background at *u*th element is given by:

(12) $$x_{u}\left(t\right)=s_{u}\left(t-\tau_{u}\left(\theta_{d},\theta_{s}\right)\right)+n_{u}\left(t\right)$$

As demonstrated in Section 2.1, the acoustic field in the DAZ is mostly contributed by the D and S1B0 arrivals. If the analytical signal of s(t) can be expressed as $\tilde{s}\left(t\right)$, the received signal can be simplified as:

(13) $$\tilde{s}\left(\theta_{d},\theta_{s},k\right)=\left[\begin{array}{c} s_{d}\\ \begin{array}{c} s_{d}exp\left\{-jkdcos\theta_{d}\right\}\\ \begin{array}{c} s_{d}exp\left\{-j2kdcos\theta_{d}\right\}\\ \vdots \end{array} \end{array}\\ s_{d}exp\left\{-j\left(M-1\right)kdcos\theta_{d}\right\} \end{array}\right]+\left[\begin{array}{c} s_{s}\\ \begin{array}{c} s_{s}exp\left\{-jkdcos\theta_{s}\right\}\\ \begin{array}{c} s_{s}exp\left\{-j2kdcos\theta_{s}\right\}\\ \vdots \end{array} \end{array}\\ s_{s}exp\left\{-j\left(M-1\right)kdcos\theta_{s}\right\} \end{array}\right]$$

where *d* is the distance between two neighboring hydrophones, *k* is the wavenumber, and the first hydrophone is regarded as the reference element. The weight vector of the beamformer is given by:

(14) $$w\left(\theta_{0}\right)={\left[1,exp\left\{jkdcos\theta_{0}\right\}\cdots ,exp\left\{j\left(M-1\right)kdcos\theta_{0}\right\}\right]}^{T},\theta_{0}\epsilon \left(\theta_{1},\theta_{2}\right)$$

Accordingly, the array output can be written as:

(15) $$y\left(t\right)=Re\left\{w^{H}\tilde{x}\left(t\right)\right\}$$

Since $\theta_{d}$ would not absolutely be equal to $\theta_{s}$, $w\left(\theta_{0}\right)$ could not absolutely eliminate the waveform distortion between arbitrary pairs of hydrophones.

Then, the correlation coefficients of the signals and the associated noise at the *u*th and *v*th element, respectively, can be expressed as:

(16) $${\left(\rho_{s}^{\prime }\right)}_{uv}=\frac{Re\left[\bar{s_{u}s_{v}^{*}e^{j\left(v-u\right)kdcos\;\theta_{0}}}\right]}{\sqrt{\bar{{\left|s_{u}\right|}^{2}}}\sqrt{\bar{{\left|s_{v}\right|}^{2}}}}$$

(17) $${\left(\rho_{n}^{\prime }\right)}_{uv}=\frac{Re\left[\bar{n_{u}n_{v}^{*}e^{j\left(v-u\right)kdcos\;\theta_{0}}}\right]}{\sqrt{\bar{{\left|n_{u}\right|}^{2}}}\sqrt{\bar{{\left|n_{u}\right|}^{2}}}}$$

Accordingly, the *OAG* is given by:

(18) $$OAG=10\;lg\frac{\sum_{u}^{M}\sum_{v}^{M}{\left(\rho_{s}^{\prime }\right)}_{uv}}{\sum_{u}^{M}\sum_{v}^{M}{\left(\rho_{n}^{\prime }\right)}_{uv}}$$

The *OAG* derived from Equation (18) could be used to estimate the performance of the vertical line array.

### 3.3. Definitions of the Signal Gain and the Noise Gain

For isotropic Gaussian noise, the sum of the correlation coefficients between all pairs of hydrophones $\sum_{i}^{M}\sum_{j}^{M}{\left(\rho_{g}\right)}_{ij}$ is equal to *M*, where *M* denotes the element number.

Accordingly, we define the signal gain (SG) of the array as the array gain in isotropic Gaussian noise conditions [18], i.e.,

(19) $$SG=10\;lg\frac{\sum_{u}^{M}\sum_{v}^{M}{\left(\rho_{s}^{\prime }\right)}_{ij}}{\sum_{u}^{M}\sum_{v}^{M}{\left(\rho_{g}^{\prime }\right)}_{ij}}=10\;lg\frac{\sum_{u}^{M}\sum_{v}^{M}{\left(\rho_{s}^{\prime }\right)}_{ij}}{M}$$

If the noise gain (NG) is defined in the same way, i.e., with the isotropic Gaussian noise as a reference, we have:

(20) $$NG\left(\theta \right)=10\;lg\frac{\sum_{u}^{M}\sum_{v}^{M}{\left(\rho_{n}^{\prime }\right)}_{ij}}{\sum_{u}^{M}\sum_{v}^{M}{\left(\rho_{g}^{\prime }\right)}_{ij}}=10\;lg\frac{\sum_{u}^{M}\sum_{v}^{M}{\left(\rho_{n}^{\prime }\right)}_{ij}}{M}$$

Therefore, the *OAG* can be expressed as:

(21) OAG=SG−NG

For a signal field that consists of a single plane wave that is normally incident on the array, we have:

(22) $$max_{\theta }\left[\sum_{u}^{M}\sum_{v}^{M}{\left(\rho_{plane\;wave}^{\prime }\right)}_{uv}\right]=M^{2}$$

Accordingly, the SG reduces to the well-known 10 *l*og*M* dB:

(23) $$SG_{plane\;wave}=10\;lg\frac{M^{2}}{M}=10\;lgM$$

It is worthwhile to note that for isotropic Gaussian noise background, there will be:

(24) $$NG_{Gaussian}=10\;lg\frac{M}{M}=0$$

while for real oceanic noise, the result of *NG* depends on the directionality and correlation of the noise. The result need not be zero and could even be a negative gain, which is often neglected. A positive *NG* will degrade the overall array performance, and a negative *NG* will enhance it, which can be seen from Equation (21) in which the noise gain is subtracted from the signal gain to give the array gain. This additional “noise” gain can be important because it can contribute to an array gain that is larger than 10 *l*og*M* dB.

## 4. Analysis of the Signal Gain and Noise Gain Based on the DAZ and RAP

### 4.1. Analysis of the Signal Gain Based on the Vertical Directionality and Correlation

**Figure 6:**
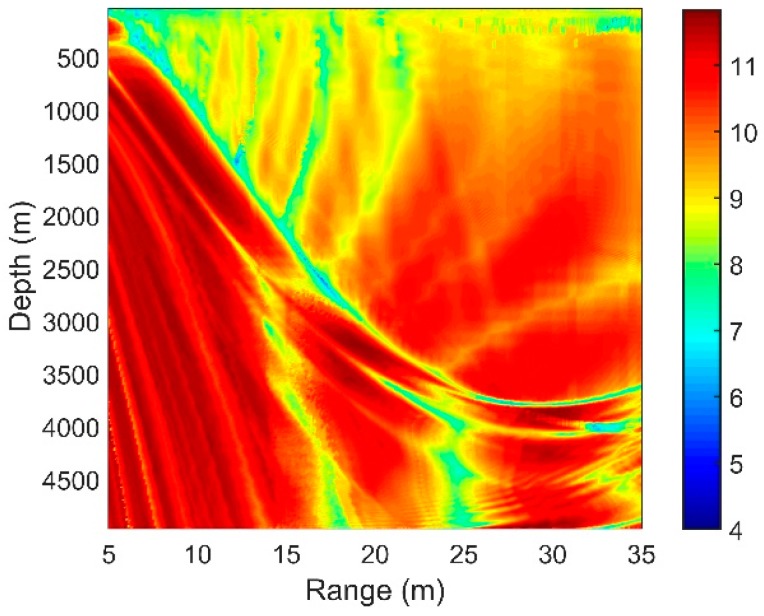
The SG with different array locations for a source at a 100-m depth.

The spatial gain distribution is significant in designing sonar equipment and determining the performance and deployment of the array. According to Section 3.3, the spatial gain obtained through CBF can be traced to the correlation coefficients of the signals and noise associated with the signals. We consider a deep ocean with the same acoustic parameters in Figure 1a. The target is at the depth of 100 m, radiating a sweeping frequency signal with the central frequency of 100 Hz and a bandwidth of 50 Hz. The vertical line array has an equal spacing of 15 m (λ/2) and contains 16 elements. By using BELLHOP model, we can generate a two-dimensional mesh in which the received signal of every point can be simulated based on ray theory, and then the SG of every point can be calculated according to Equation (19). Figure 6 shows the SG with different array locations.

In the DAZ, the SG is large along the ray path where the constructive interference between D and S1B0 appear. As demonstrated in Section 2.1, the acoustic fields in these areas are mainly composed of D and S1B0, whose arrival angles are approximately equivalent on every element of the array. Hence, the received signal’s phase difference between two neighbouring elements is almost fixed. Since the array elements are almost in the same phase plane with the proper phase shift, the SG is relatively large but could still not exceed 10 *log16* dB. At the same time, the SG declines slightly along the path where the destructive interference between D and S1B0 appear. Other than D and S1B0, S0B1 and S1B1 also contribute to the sound field here. The received signals with the proper phase shift by CBF still has large phase distortion due to the large differences in the multipath arrival angles. Hence, the correlations between the signals are relatively poor.

It is worthwhile to note that the theoretical solution of the vertical correlation coefficient based on the ray model can also be used to interpret the decline. According to [4], the vertical correlation coefficient in the frequency domain can be written as:

(25) $$\rho^{\prime }\left(z,z+\Delta z\right)=\frac{Re\left[\int_{\omega_{1}}^{\omega_{2}}s_{z}\left(\omega \right)s_{z+\Delta z}^{*}\left(\omega \right)e^{e^{jk\Delta zcos\;\theta_{0}}}d\omega \right]}{\sqrt{\int_{\omega_{1}}^{\omega_{2}}{\left|s_{z}\left(\omega \right)\right|}^{2}d\omega \int_{\omega_{1}}^{\omega_{2}}{\left|s_{z+\Delta z}\left(\omega \right)\right|}^{2}d\omega }}$$

where $s_{z}\left(\omega \right)$ and $s_{z+\Delta z}\left(\omega \right)$ are the complex spectra of the acoustic signals $s_{z}\left(t\right)$ and $s_{z+\Delta z}\left(t\right)$, respectively. In addition, *ω*_1_ and *ω*_2_ are the lower and upper angular frequencies of the filtered acoustic signals, respectively. When the acoustic field can be written simply as the sum of the contributions of D and S1B0, we have:

(26) $$p_{z}\left(\omega \right)=A_{Z}^{D}\left(\omega \right)e^{j\omega t_{z}^{D}}-A_{Z}^{S}\left(\omega \right)e^{j\omega t_{z}^{S}}$$

where $A_{Z}^{D}$ and $t_{z}^{D}$ are the amplitude and arrival time of D, respectively, and $A_{Z}^{S}$ and $t_{z}^{S}$ are those for S1B0. We assume that $A_{Z}^{D}\left(\omega_{0}\right)=A_{Z}^{s}\left(\omega_{0}\right)$, where $\omega_{0}$ is the center angular frequency. Therefore, ρ(z,z+Δz) in Equation (25) can be simplified as:

(27) $$\rho^{\prime }\left(z,z+\Delta z\right)=\left|\cos\left[\frac{\omega_{0}}{2}\left(\Delta t_{z}-\Delta t_{z+\Delta z}\right)\right]\right|$$

Swhere $\Delta t_{z}=t_{z}^{S}-t_{z}^{D}$ and $\Delta t_{z+\Delta z}=t_{z+\Delta z}^{S}-t_{z+\Delta z}^{D}$ are the relative time delays of D and S1B0 for the receiver depths of z and z+Δz, respectively.

Seen from Equation (27), the vertical correlation coefficient has an oscillatory period depending on both the center angular frequency $\omega_{0}$ and the differences in the relative time delays at two different element depths. As shown in Figure 3, if the location is dominated by D and S1B0, whose relative time delays at different depths remain almost constant, the correlation coefficients between pairs of hydrophones can be close to 1. Hence, the SG in these areas will be high.

Overall, if the acoustic field is dominated by the other two types of arrivals, the vertical correlations can also be predicated by Equation (27), with $\Delta t_{z}$ being the relative time delay of the other two types of arrivals. Next, we consider the locations where the acoustic field is dominated by D, S1B0 and S0B1. From Figure 3, the lines of the arrival times of D and S1B0 are approximately parallel, and thus, we can regard the arrival times of D as the reference. The relative time delays of D and S0B1 decrease with an increase in the element number. Consequently, the correlation coefficients between pairs of hydrophones will be oscillatory, and the SG in these areas will decline.

For the RAP, the SG is large in most of the space other than the array locations for which the range of the sources is 24 to 28 km. This finding is mainly caused by the variety of structure in the multipath arrivals.

**Figure 7:**
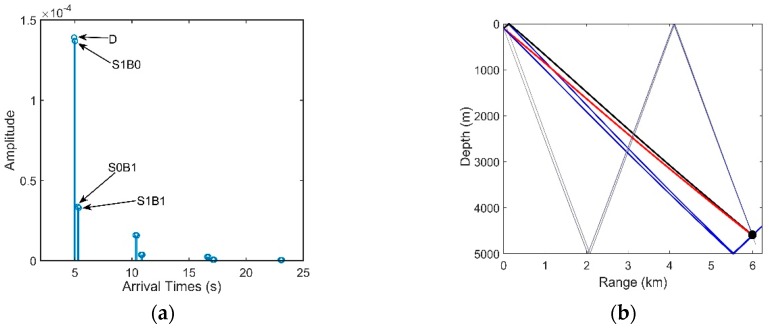

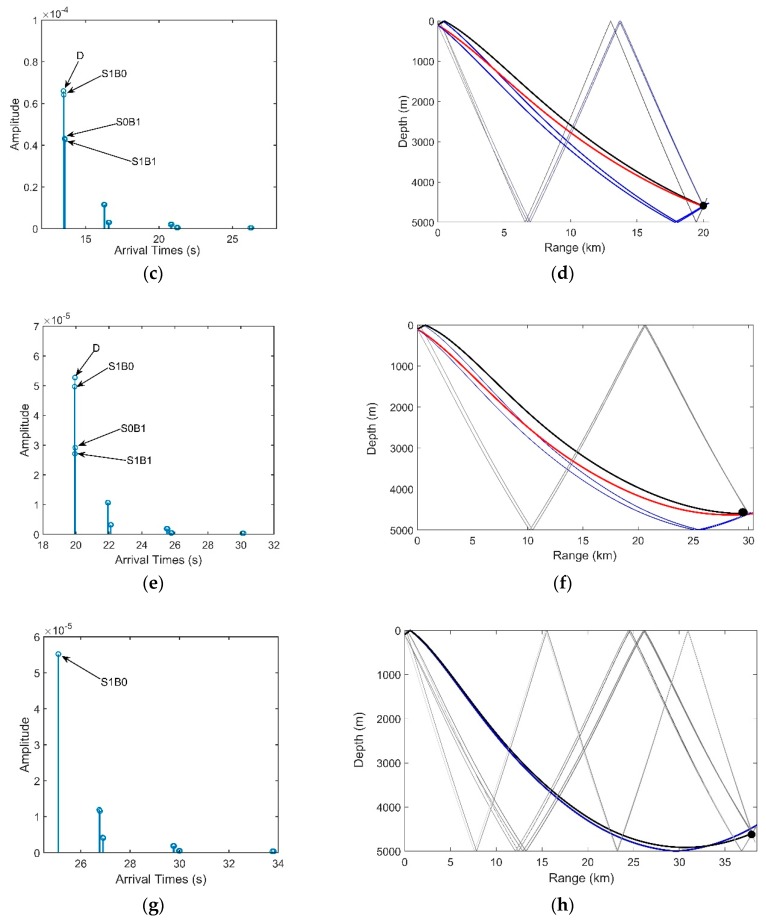
Arrival structures for the horizontal ranges of (**a**,**b**) 6 km, (**c**,**d**) 20 km, (**e**,**f**) 30 km and (**g**,**h**) 38 km.

The arrival structures of the eigenrays from a 100-m deep source to the hydrophone at a depth of 4550 m in a range-independent ocean are shown in Figure 7. Four horizontal ranges of the source are assumed to be 6, 20, 30, and 38 km, respectively. Other acoustic parameters are the same as those in Figure 1a. The rays that penetrate into the bottom are neglected, and thus, the acoustic properties of the half-space are chosen to simulate the sediment layer in the deep ocean. The frequency for the simulation is 150 Hz. At the range of 6 km, only D and S1B0 are significant. At this range, rays penetrate into the bottom with large grazing angles, and thus, major energy is attenuated in the substrate [17]. When the source is fixed at 20 km in the range, in addition to D and S1B0, the S0B1 and S1B1 become significant. As the rays penetrate into the bottom with smaller grazing angles, more energy is reflected into the water. Therefore, the SG declines to approximately 7–8 dB when the source is located at 19–25 km because there is a relatively large difference in the multipath arrival angles between two groups of multipath signals (“D, S1B0” and “S0B1, S1B1”) with almost the same amount of energy. B1S0 is weak in this range due to the large grazing angle on the bottom and the additional geometric attenuation. For the source at 30 km, two new types of rays become significant. D, S0B1, and S1B1 are strongly bent, which indicates that they are close to being cut off by the gradient of the SSP. From Figure 7f, the D, S0B1 and S1B1 are bent to approximately 0° in such a way that the arrival angles of these rays are approximately the same. The waveform distortion between pairs of hydrophones become small due to having only a small difference in the multiple arrival structures, and hence, a relatively high SG can be achieved through CBF. According to the definition of the RAP introduced in Section 2.2, this source range is close to the edge of the RAP. At the source range of 38 km, the three types of rays are cut off, and the S1B0 exhibits strong bending; hence, this range is so long that it exceeds the region of the RAP.

### 4.2. Results of the Signal Gain

**Figure 8:**
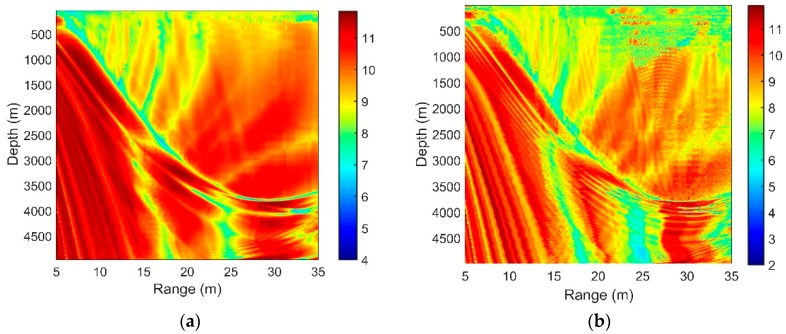
SG with different array locations for the center frequencies of (**a**) 150 Hz and (**b**) 450 Hz.

**Figure 9:**
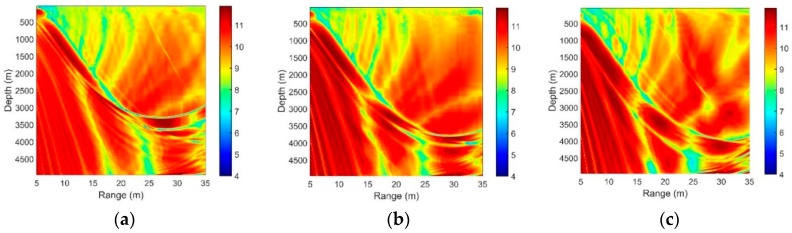
SG with different array locations for the sources at the depths of (**a**) 50 m, (**b**) 100 m and (**c**) 200 m.

**Figure 10:**
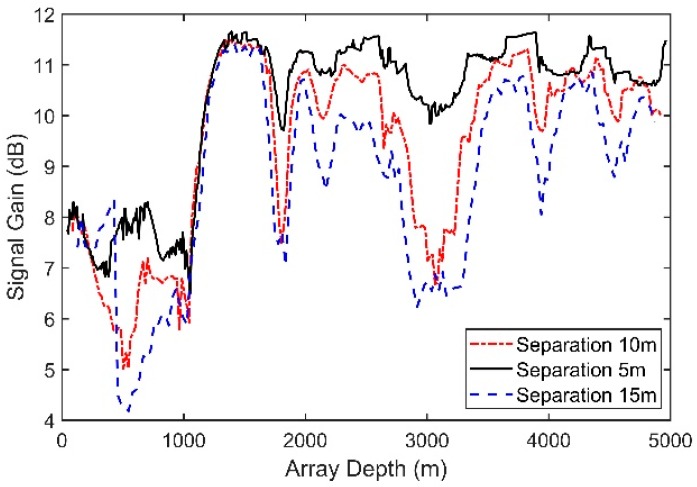
SG for arrays with different separations of neighboring hydrophones.

**Figure 11:**
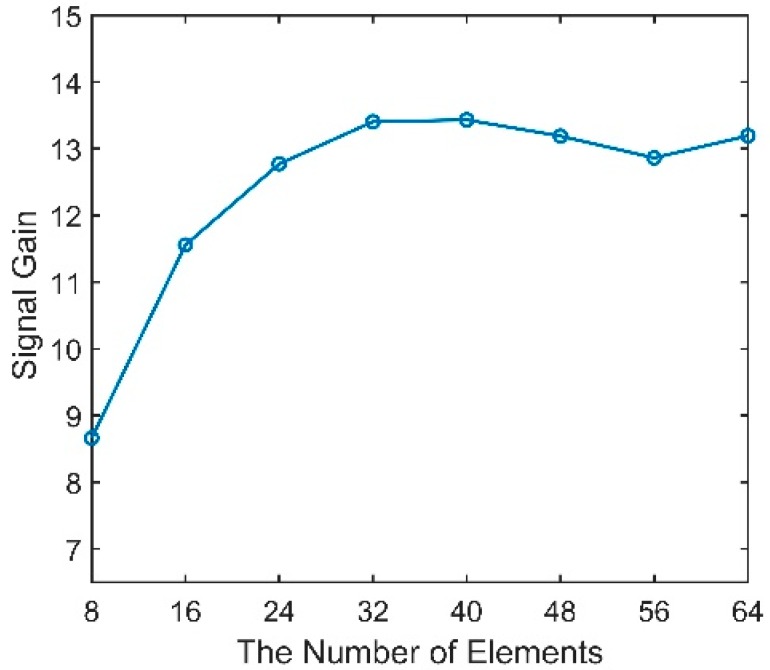
The variation in the SG with increasing numbers of hydrophones.

Figure 8, Figure 9, Figure 10 and Figure 11 present the SG under different circumstances, and the acoustic parameters are the same as those in Part 2.

Figure 8a,b show the SG in different source frequencies. The source depths are both 100 m, and the separation of the hydrophones is λ/2. We can see that as the source frequency increases, the interference striations are closer. With increasing acoustic frequency, the SG declines moderately.

Figure 9a–c present the SG with the source depths of 50 m, 100 m and 200 m, respectively. According to the *Lloyd-mirror* interference pattern [16], by simplifying, the received pressure of the sensor can be written as

(28) $$\left|p\right|\approx \frac{2}{\sqrt{r^{2}+z_{r}^{2}}}\sin\frac{\omega z_{s}z_{r}}{c\sqrt{r^{2}+z_{r}^{2}}}$$

where $z_{r}$ is the fixed depth of the hydrophone, and $z_{s}$ is the source depth, while r denotes the horizontal distance between the source and receiver. Equation (28) means that the frequency-dependent interference period decreases as the radiated frequency or source depth increases. Figure 9a–c all match well with the Equation (28).

It can be found that the source depth (shallow source) has no substantial influence on the SG of the vertical line array achieved by the CBF.

Figure 10 presents the SG for arrays with different separations of neighboring hydrophones. The vertical line array contains 16 elements, and the source radiates a 150-Hz signal with a depth of 100 m and a range of 10 km. With an increase in the element separation, the SG declines moderately. This trend occurs mainly because a high ratio of the element separation to the wavelength will lead to a greater difference between the multipath structures in different hydrophones.

Figure 11 shows the variation in the SG with increasing numbers of hydrophones. Unlike the SG, which will increase logarithmically as the number of elements increases in a horizontal array, after increasing to a certain level, the SG here grows slowly and even begins to decrease. Similarly, this trend occurs mainly because a larger array aperture will lead to a greater difference between the multiple structures in the different hydrophones and, hence, to a lower coherence between signals. Therefore, simply increasing the number of elements in the vertical array has little effect.

**Figure 12:**
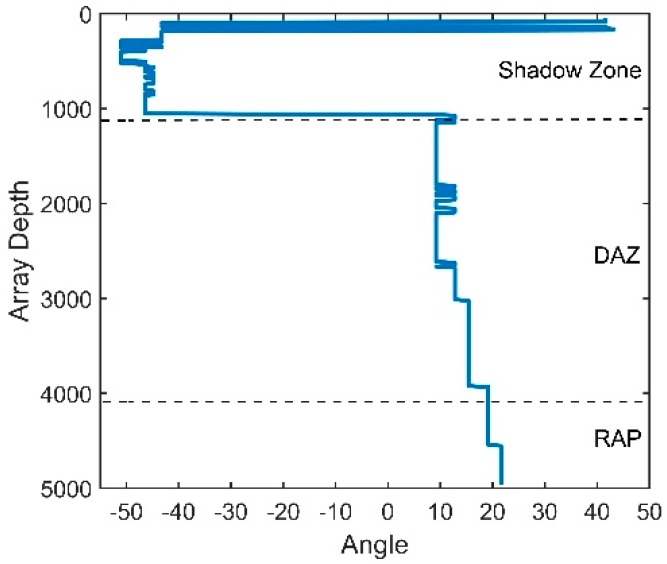
Vertical directionality of signals with different array depths.

We define the arrival angles that correspond to the maximum angular response of the CBF as the vertical directionality of the acoustic signal. The 100 m-deep source is fixed in the range of 10 km, radiating a sweeping frequency signal with a center frequency of 150 Hz and a bandwidth of 50 Hz. The vertical array contains 16 elements with an equal spacing of 5 m, and the other parameters stay unchanged. The vertical directionality of the signals with different array depths is shown in Figure 12. It can be seen that from the top of the DAZ to the base of the RAP, the elevation angles keep increasing and almost equal the arrival angle of D. As illustrated above, under this source range, the received signals in the DAZ and RAP are dominated by D and S1B0. The direction of the acoustic signal in the shadow zone varies almost without a law because of its unstable multipath arrivals.

### 4.3. Vertical Directionality and Correlation of the Noise

Ocean ambient noise is a type of acoustic background, which constantly exists in the ocean and is produced by a number of different types of noise sources. Wind-driven and distant shipping noise sources contribute to the total noise field in the DAZ and RAP of the deep ocean [13]. In a real ocean, the ambient noise is not incoherent and anisotropic, and thus, both the directivity and the correlation are important for the NG.

The directional density function represents the noise power that is incident at a point receiver in the ocean as a function of the arrival angle. Assuming spherical-polar coordinates, the directional density function can be written as F(θ,φ), where θ is the polar angle measured from the zenith, and φ is the azimuthal angle. A convenient normalization, introduced by Cox [9], equates the total noise power integrated over all angular space to 4π:

(29) $$\int_{0}^{2\pi }\int_{0}^{\pi }F\left(\theta ,\varphi \right)sin\theta d\theta d\varphi =4\pi$$

For the case of surface-generated noise, where the sources are distributed uniformly (statistically) across the sea surface, the directional density function is independent of the azimuth, in which case the normalization in Equation (29) reduces to:

(30) $$\int_{0}^{\pi }F\left(\theta \right)sin\theta d\theta =2$$

where F(θ) represents the power directivity of the noise in the vertical direction, integrated over the azimuth.

Cox proposed that any homogeneous noise field could be characterized by the directional distribution of the noise power. His famous coherence function for vertical aligned sensors is:

(31) $$C\left(kd\right)=\frac{1}{2}\int_{0}^{\pi }F\left(\theta \right)e^{-j\left(kd\right)cos\theta }sin\theta d\theta$$

where d stands for the separation between two hydrophones.

For the distant shipping noise, the dual peaks structure [14], in which the peaks are approximately symmetrical with respect to the horizontal direction, can be observed in the vertical directionality pattern. This directionality result was induced by upward and downward acoustic rays from the DSC and along the DSC or convergence zone path, because acoustic rays can reverse at the surface conjugate depth. According to [14], the directional density function of the distant shipping noise is developed on the basis of the Von Mises circular distribution:

(32) $$F\left(\theta \right)=a_{0}e^{\mu cos2\left(\theta -\theta_{0}\right)-\mu }+a_{1}e^{\mu cos2\left[\theta -\left(\pi -\theta_{0}\right)\right]-\mu },\theta ,\theta_{0}\epsilon \left[0,\pi \right]$$

To improve the intuitiveness, Equation (32) is shifted from the angle interval [0,π] to the interval $\left[-\frac{\pi }{2},\frac{\pi }{2}\right]$ in this paper, and then, it will be rewritten as:

(33) $$F\left(\theta \right)=a_{0}e^{\mu cos2\left(\theta -\theta_{0}\right)-\mu }+a_{1}e^{\mu cos2\left(\theta +\theta_{0}\right)-\mu },\theta ,\theta_{0}\epsilon \left[-\frac{\pi }{2},\frac{\pi }{2}\right]$$

where $\pm \frac{\pi }{2}$ denotes the vertical endfire directions. The positive and negative angles represent the downward and upward acoustic rays arriving at the array, correspondingly.

This expression shows dual peak at the angles $\theta_{0}$ and $-\theta_{0}$. The real parameters $a_{0}$ and $a_{1}$ decide the height of the peaks, and they will be symmetrical with respect to the horizontal axis when $a_{0}=a_{1}$. In general, the power is lower along the upward rays than along the downward rays in the noise directionality pattern, i.e., $a_{0}>a_{1}$. This finding occurs mostly because the ocean acoustic channel was an incomplete channel with a lack of surface conjugate depth. The acoustic rays could not reverse completely but were reflected by the bottom with reflection losses. The parameter μ decides the peak’s width, and the width becomes narrower when μ increases.

**Figure 13:**
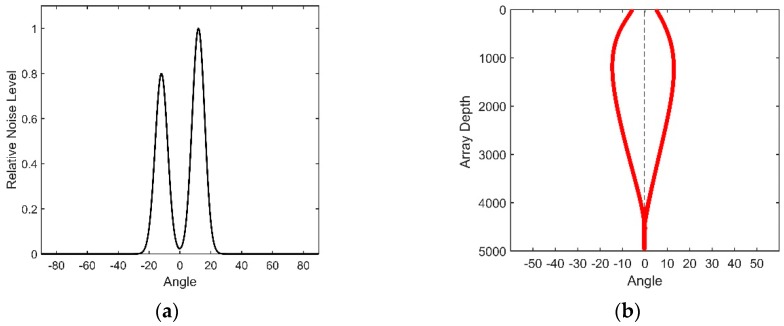
(**a**) Shipping noise directional density at the depth of the DSC; (**b**) Shipping noise directional density for all depths.

The directional density function specified by Equation (33) is plotted in Figure 13. The value of $\theta_{0}$ is set to 12°; μ is set to 60, and $a_{0}=1$, $a_{1}=0.8$.

The directional density of the shipping noise versus the depth has been generated in [14], as shown in Figure 13b. This figure shows that the directional density depends on the depth. The black line in Figure 13b means the horizontal direction. This type of dual-homed phenomenon can be explained by ray theory. Both of the upgoing and downgoing rays exist at the receiver’s station, and these two types of rays are symmetric about the horizontal. According to Snell’s law, acoustic rays bend to the depth where the sound has the lower speed. Therefore, the notch has the maximum width at the DSC depth. When reaching the surface conjugate depth, the rays start to reverse, and the angles of the rays versus the horizontal approach 0° gradually; thus, the notch disappears below the conjugate depth.

According to Cox’s theory, by substituting Equation (33) into Equation (31), the vertical coherence function takes the form of:

(34) $$C\left(kd\right)=\frac{1}{2}\int_{-\frac{\pi }{2}}^{\frac{\pi }{2}}\left\{a_{0}e^{\mu cos2\left(\theta -\theta_{0}\right)-\mu }+a_{1}e^{\mu cos2\left(\theta +\theta_{0}\right)-\mu }\right\}e^{-j\left(kd\right)cos\theta }sin\theta d\theta$$

**Figure 14:**
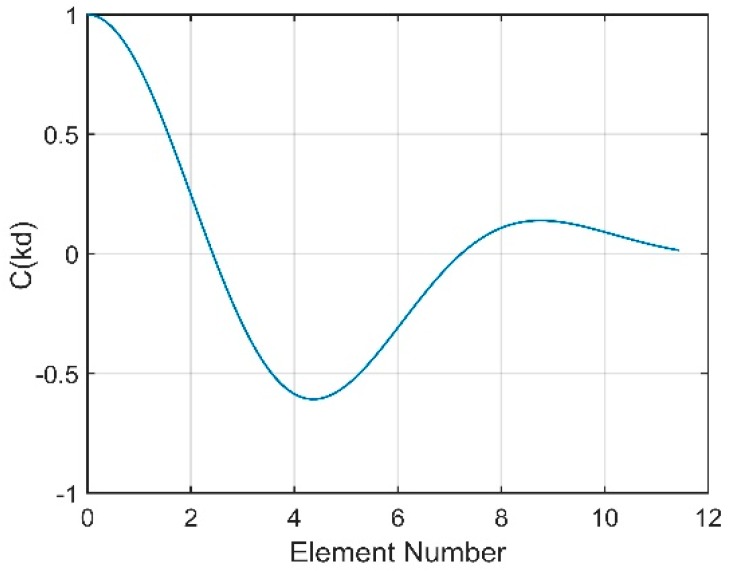
Vertical correlation coefficient of the shipping noise at the frequency of 350 Hz.

As illustrated above, positive and negative angles represent the downward and upward acoustic rays that arrive at the array, correspondingly. The vertical correlation coefficient of the distant shipping noise field at 350 Hz is presented in Figure 14. It has been verified in [14] that the experiment result matches quite well with this theoretical coherence.

**Figure 15:**
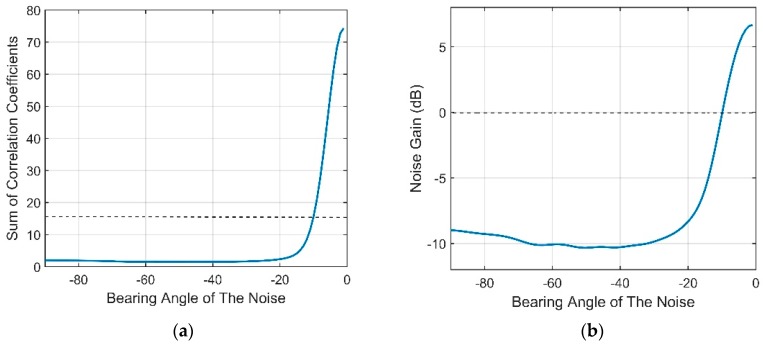
(**a**) The sum of the correlation coefficients for different noise directions; (**b**) the NG for different noise directions.

According to Equation (20), the NG is related to the sum of the correlation coefficients between the noise contributions of all pairs of hydrophones. Figure 15a presents the sum versus various values of $\theta_{0}$. The decrease in $\theta_{0}$ implies that the interval between the two peaks decreases. When $\theta_{0}$ decreases to almost 0°, the noise will be incident on the vertical array from directions closer to the horizontal, which is the array’s natural steering angle. Seen from Figure 15a, the coherence radius is farther from the origin when $\theta_{0}$ decreases. This finding illustrates that the closer the steering angle of array is to the direction of the noise, the larger the coherence radius will be. Consequently, the correlation coefficients of all pairs of hydrophones will be higher, which ultimately causes a higher NG. The variation in the NG with $\theta_{0}$ is presented in Figure 15b. When $\theta_{0}$ decreases to 0°, which is the natural steering angle of the array, the NG will reach 6.7 dB. In contrast, a negative NG, which could enhance the array gain, can be achieved when the array’s steering angle is far away from the direction of the noise. This finding shows that the NG will be positive if the steering angle of the CBF is within approximately ±10° of the noise direction.

According to [13], the spatial correlation coefficients of the wind-driven noise field were approximately consistent with the results of the Cron/Sherman (C/S) model based on the surface noise source distribution. The first zero location occurred at a half wavelength for the wind-generated noise field and C/S coherence function. Hence, for most of the wind-driven noise fields in the ocean environment, the C/S model is a good simplification for computing and modeling.

Cron and Sherman proposed a model of deep ocean ambient noise in which independent point sources were distributed uniformly in a horizontal plane beneath the sea surface by considering the ocean itself to be a semi-infinite, homogeneous half space. They assumed straight line propagation in infinitely deep water. The noise sources radiate sound with an amplitude directional pattern given by $cos^{m}\alpha$ (where *m* is a positive integer, usually taken as 1 or 2). Therefore, the vertically directional density function is:

(35) $$F\left(\theta \right)=\{\begin{array}{cc} 4mcos^{2m-1}\theta & 0\leq \theta \leq \frac{\pi }{2}\\ 0 & -\frac{\pi }{2}\leq \theta \leq 0 \end{array}$$

When *m* = 1, Equation (35) becomes:

(36) $$F\left(\theta \right)=\{\begin{array}{cc} 4cos\theta & 0\leq \theta \leq \frac{\pi }{2}\\ 0 & -\frac{\pi }{2}\leq \theta \leq 0 \end{array}$$

This result is a typical result given by the C/S model, in which the vertically directional density function does not change with the depth. It is the maximum from the zenith, and the noise density from the horizontal and the angle interval $\left[-\frac{\pi }{2},0\right]$ become zero.

By substituting Equation (36) into Equation (31), the vertical coherence function of the C/S model takes the form of:

(37) $$C\left(kd\right)=\frac{1}{2}\int_{0}^{\frac{\pi }{2}}\left(4cos\theta \right)e^{-j\left(kd\right)cos\theta }sin\theta d\theta$$

**Figure 16:**
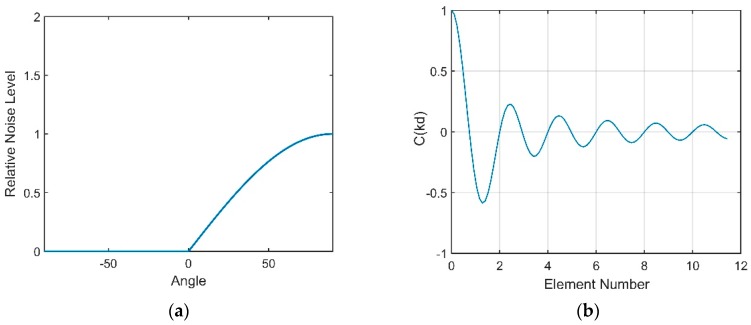
(**a**) Vertical directional density of the wind-driven noise at the frequency of 350 Hz; (**b**) Vertical correlation coefficient of the wind-driven noise at the frequency of 350 Hz.

Therefore, the vertical directionality and correlation coefficient of the wind-driven noise field at 350 Hz are presented in Figure 16.

**Figure 17:**
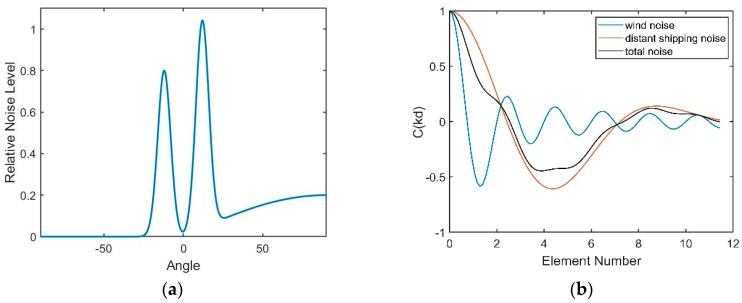
(**a**) Directional density of the total noise at the depth of the DSC; (**b**) Vertical correlation coefficients.

As demonstrated above, wind-driven and distant shipping noise sources contribute to the total noise field in the DAZ and RAP. This paper mainly considers the noise background at a wind speed of 3 m/s. The distant shipping noise reaching the vertical line array from a shallow grazing angle was the dominant noise source when the wind speed was 3 m/s. The vertical directionality and correlation coefficient of the total noise under this circumstance are presented in Figure 17, and they show good agreement with the results of our experiment and the experiment in [13]. In fact, the dual peaks structure can often be observed in the vertical directionality pattern of the noise as the presence of the DSC and bottom reflection.

We should note that by using CBF, the correlation between the signals of two elements have little to do with their spatial separation, while a smaller separation could lead to a higher correlation between the noise in the two signals. Therefore, the space between two neighboring elements should not be too small. According to the vertical correlation coefficient diagram of the frequency-depth in [13], although the location of the first zeros depends to some extent on the frequency and wind speed, the correlation coefficients under any wind speed or frequency could achieve negative values when the separation between two hydrophones is larger than approximately 10 m. It could contribute to a negative NG and enhance the performance of the array.

## 5. Analysis and Results of the Optimal Array Gain

**Figure 18:**
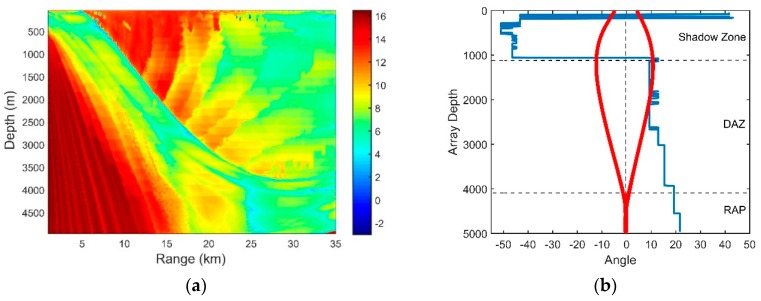
(**a**) OAG with different array locations; (**b**) Comparison of the directions of the signal and noise for all depths at a 10-km range.

The OAG can estimate the detection performance of the vertical line array effectively. According to Equation (21), we can now combine our results of SG and NG in the previous sections to see the distribution of the OAG. The higher the SG is and the more negative the NG, the higher the OAG will be. Considering a 16-element vertical line array with an equal separation of 5 m, the OAG with different array locations is presented in Figure 18a. The acoustic source is at the depth of 100 m, radiating a sweeping frequency signal with a center frequency of 150 Hz and a bandwidth of 50 Hz. The total noise consists of the distant shipping noise and the wind-driven noise when the wind speed is 3 m/s. Other acoustic parameters are unchanged.

Compared with the distribution of the SG in Figure 6, it can be seen that the OAG in the edge of the DAZ and the remote region of the RAP decreases, while those in other areas are enhanced. As demonstrated above, this finding occurs mainly because the directions of the signals and the noise in the signals in these regions are close or even matched. As shown in Figure 18b, the directivity of the signal and noise are close in the DSC. Similarly, D and S1B0 are bent to the direction near the horizontal by the sound velocity gradient beyond the range of 25 km in the RAP, and the maximum angular response of the NG also corresponds to transverse propagation here. Having approximately the same incident directions for the signal and noise leads to a positive NG. While in many other areas of the DAZ and RAP, because of the differences between their directions, a negative NG could be achieved in such a way that the OAG is relatively enhanced. In some locations, such as the regions within the range of 10 km in the RAP, the array can even achieve gains that exceed 10 *log*16 dB by CBF.

**Figure 19:**
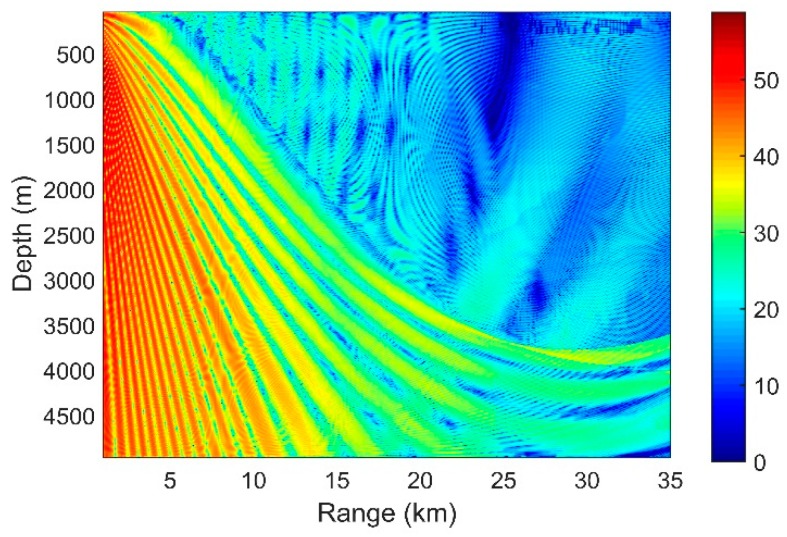
DT with different array locations.

According to the sonar equation, we can achieve the detection threshold in different locations:

(38) DT=SL−TL+AG−NL

where *DT* is the detection threshold, *SL* and *NL* are the source level and noise level respectively, *TL* denotes the transmission loss which can be calculated by BELLHOP model. For the sake of simplification, *SL* is set to a fixed value 160 dB and *NL* is set to 60 dB. Therefore, DTs in different array locations are shown in Figure 19.

It is worthwhile to note that although the OAG in the RAP 24 km away is relatively low, the output *SNR* will still be high in common cases. This result occurs because the input *SNR* in the RAP is high, and there is little oceanic noise that can access the RAP. In other words, the NL in RAP will be lower than that in other areas. Therefore, the DT in RAP in real ocean will be higher than that shown in Figure 19. In addition, although the OAG in some areas of the shadow zone can be high, the signal in the shadow zone is mainly contributed by the bottom-reflected waves which are unreliable and the TL of it is high.

## 6. Experimental Results

**Figure 20:**
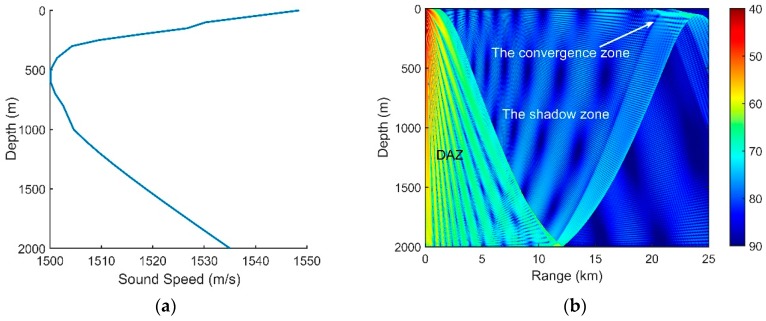
(**a**) Measured sound speed profile; (**b**) Transmission loss for a source at a 50-m depth at the frequency of 350 Hz.

An experiment was conducted in a deep ocean area of the South China Sea. The measured SSP is shown in Figure 20a; the ocean depth was approximately 2000 m, and the depth of the DSC was approximately 550 m. In this experiment, a vertical line array that consists of 16 elements with an equal spacing of 5 m was used, and the array center was fixed at a depth of approximately 432 m. In actual situations, the signal always intermingles with the oceanic ambient noise. To achieve the OAG by using Equation (21), a pure signal is required. An acoustic source radiating single frequency of 350 Hz with a high *SNR* is used in the experiment. Therefore, the output signal through a narrow bandpass filter could approximately substitute for the pure signal. In addition, the collecting noise data were filtered by a bandpass filter with a bandwidth of 100 to 1 k Hz. The experimental data were measured in two different source-receiver ranges.

First, the source with a depth of 50 m was located at the horizontal range of 2.4 km. Figure 20b is the transmission loss. The array was located in the DAZ, and thus, the received signal was mainly composed of D and S1B0.

**Figure 21:**
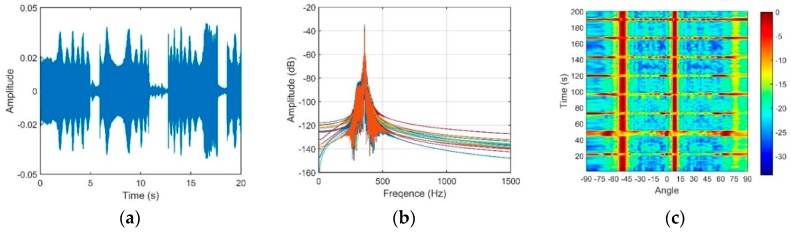
(**a**) Signal waveform; (**b**) Signal frequency through a narrow bandpass filter; (**c**) Beam output of the signal.

**Figure 22:**
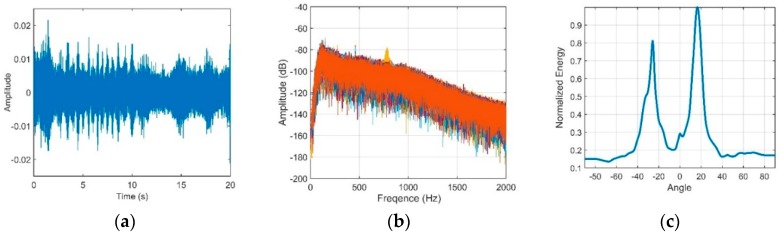
(**a**) Noise waveform; (**b**) Noise frequency through a 100 Hz to 1 kHz bandpass filter; (**c**) Directionality of the noise.

The vertical structures of the signal and noise through the CBF are shown in Figure 21 and Figure 22, respectively.

It can be seen in Figure 21c that the maximum of the beam output of the received signal corresponds to the elevation angle of 11.6°, which is close to the arrival angles of D and S1B0. B1S0 is weak in this range due to the large grazing angle on the bottom and the additional geometric attenuation. The target that corresponds to the elevation angle of −50° is a false target. This phenomenon occurs when the separation between two neighboring elements is larger than half of the wavelength [19]. The angle difference between the true angle and false angle is given by:

(39) $$\sin\left(\Delta \theta \right)=\frac{\lambda }{d}=\frac{c}{fd}$$

According to Equation (39), Δ*θ* = 61.2°. This finding is consistent with the results shown in Figure 21c. The true elevation angle that we need corresponds to the angle of 11.6°, and it coincides with the theory proposed in Part 3.2.

**Figure 23:**
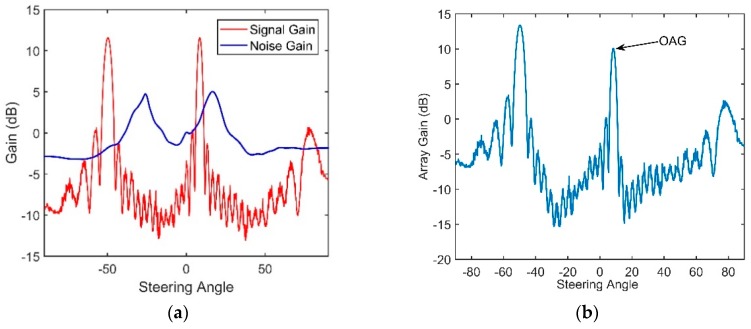
(**a**) The variation in the SG and NG with steering angles of CBF and (**b**) The variation of Optimal array gain with steering angles of CBF under a 2.4-km source range.

Figure 21c, shows the dual-horned structure in the vertically directional density. The elevation angles of the two peaks are approximately −25.7° and 16.5°. The variations in the SG and NG with different steering angles after beamforming are shown in Figure 23a. As demonstrated above, the directionality of the NG matches well with that of the noise. The NG that corresponds to relatively steep angles and the NG near horizontal are negative, and they can enhance the performance of the array. The maximum angular response of the SG corresponds to the arrival angles of D and S1B0, which are approximately 11.6°. Seen from Figure 23b, ultimately the OAG whose value is 10.08 dB is achieved at the elevation angle of 11.6°. These findings show good agreement with our simulation. For the vertical array located within the range of the DAZ, by using the CBF, the correlation coefficients between pairs of hydrophones will be high. At the same time, a lower NG can be achieved if the steering angle of the CBF avoids the main incident direction of the noise because it contributes to low correlations between the noise components of the elements. In our experiment, a negative NG can be achieved when the space between two neighboring hydrophones is 5 m, but the negative values are often limited when the CBF is adopted. Seen from Figure 23a, the NG versus 11.6° is a positive value, which is 1.58 dB, and it will degrade the OAG. As illustrated in Part 5, the main reason is that the main incident direction of the noise is close to the arrival angle of D and S1B0 near the region of the DSC. Therefore, the value of OAG under this condition does not exceed the ideal array gain 10 *log*16 dB. This is consistent with our simulation.

**Figure 24:**
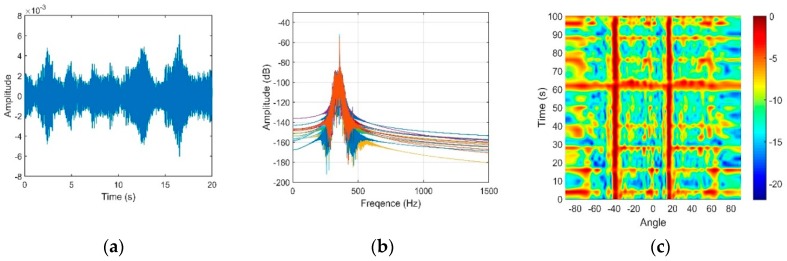
(**a**) Signal waveform; (**b**) Signal frequency through a narrow bandpass filter; (**c**) Beam output of the signal.

**Figure 25:**
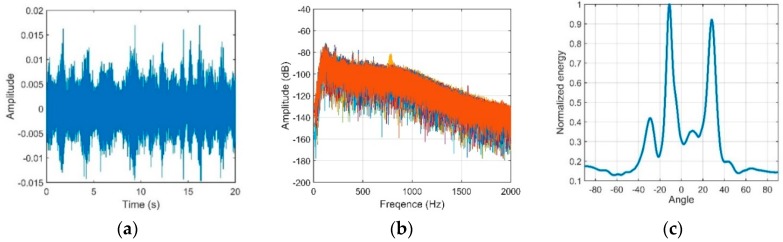
(**a**) Noise waveform; (**b**) Noise frequency through the 100 Hz to 1 kHz bandpass filter; (**c**) Directionality of the noise.

Similarly, when the source with a depth of 10 m was located at the horizontal range of 4.0 km, we present the vertical structures of the signal and noise in Figure 24 and Figure 25, respectively.

The dual-horned structure in the vertical directional density of the noise can also be seen from Figure 25c, and the elevation angles of the two peaks are approximately −11.1° and 28.1°. These findings show good agreement with the results in Figure 22.

**Figure 26:**
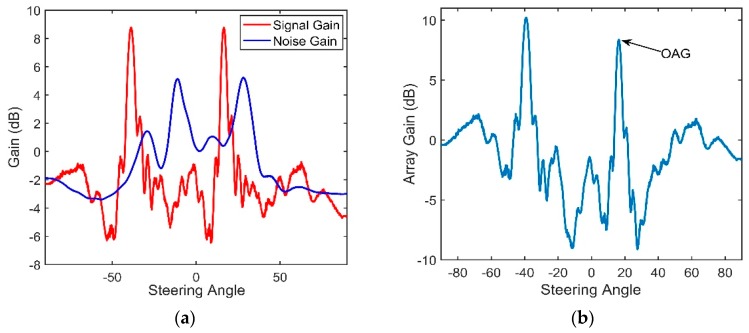
(**a**) The variation in the SG and NG with steering angles of CBF, and (**b**) The variation in the optimal array gain with steering angles of CBF under a 4-km source range.

The OAG under these two source-receiver ranges are presented in Table 1. Because the signals that we used to calculate the gains are still mixed with a small amount of noise, the actual OAG will be slightly higher than those achieved in the table. It can be seen that the OAG at the 4-km source-receiver range decreases to 8.4 dB. This finding can be interpreted through Figure 24. Compared with Figure 21, the input *SNR* in Figure 24 is relatively low. The signals collected under this condition was not pure signals and intermingled with relatively more ambient noise. As shown in Figure 26a, the SG corresponds to the maximum angular response decreases to 9.1 dB. At the same time, because the receiver depth is near DCS, the OAG achieved in these two ranges are both degraded by the NGs. The results from Figure 23, Figure 24, Figure 25 and Figure 26 verify the effectiveness of our simulation analysis.

## 7. Conclusions

The array gain is a key parameter in evaluating the detected performance of the vertical line array. The proposed method of gain analysis provides analysis methods for a vertical array in the deep ocean under other conditions with references. In this work, the experimental results were consistent with the simulation analysis on the DAZ in the deep ocean. Therefore, several conclusions are summarized, as follows:

1. The OAG can be given by an equation in which the NG is subtracted from the SG, and hence, a high SG and a negative NG can enhance the OAG of the array. The SG and NG are related to the correlation coefficients of the signals and their noise, respectively.
2. The signal in the DAZ is dominated by D and S1B0, and there is little difference between their arrival structures. Therefore, a relatively high correlation between the signals of different elements can be achieved through the CBF, which contributes to a high SG directly. For the RAP, the values of the SG in the RAP changes with the source-receiver range because of the variety of structures in the multipath arrivals.
3. The source depth (shallow source) has no substantial influence on the SG. With an increase in the element separation or source frequency, the SG declines moderately. This trend occurs mainly because a high ratio of the separation to the wavelength will lead to a greater difference between the structures of multipath arrivals in different hydrophones. Increasing numbers of elements in the vertical array cannot keep enhancing the SG because a larger array aperture will lead to a greater difference between the multiple structures in different hydrophones, and hence, it will lower the correlation between signals.
4. A dual peaks structure can often be observed in the vertical directionality pattern of the noise because of the presence of the DSC and bottom reflection, and the two peaks are always near and approximately symmetrical in the horizontal direction. When the directions of the signal and noise are close, the beamformer only will enhance the correlation of the signals but will also enhance that of the noise. This relationship will lead to a relatively high NG and hence degrades the performance of the array.
5. Under the condition of a typical SSP in the deep ocean, an OAG at a middle distance of the DAZ and RAP can be high and even exceed 10 *logN* dB, while at the edge of the DAZ and in a remote region of the RAP, it will decrease. The input SNR in the RAP is always high because there is little oceanic noise that can access the RAP. Therefore, the output SNR in the RAP will still be high even though the OAG in some areas of it is relatively low. Although the OAG in some areas of the shadow zone can be high, the signal in the shadow zone is mainly contributed by the bottom-reflected waves which are unreliable and the TL of it is high.

## Figures and Tables

**Table 1:** The values of the optimal array gain under the above two experimental conditions.

Source Range	2.4 km	4 km
OAG (dB)	10.08	8.4
Steering angle (degrees)	11.6	8.7
